# The first attack of multiple sclerosis presented immediately after voluntary and intensive weight loss: A case series

**Published:** 2017-01-05

**Authors:** Sama Bitarafan, Kiana Amani, Mohammad Ali Sahraian, Payam Sarraf, Danesh Soltani, Abdorreza Naser Moghadasi, Mohammad Hossein Harirchian

**Affiliations:** 1 Iranian Center of Neurological Research, Neuroscience Institute, Tehran University of Medical Sciences, Tehran, Iran; 2 Multiple Sclerosis Research Center, Neuroscience Institute, Tehran University of Medical Sciences, Tehran, Iran

**Keywords:** Multiple Sclerosis, Weight Loss, Intensive, Diet

There are many studies have reported that nutritional deficiencies (macro and micronutrients) are involved in the etiology of multiple sclerosis (MS).^1^

Some neurological complications such as polyneuropathy and optic neuropathy that are common in MS have been observed in consequential severe weight loss (WL) after bariatric surgery. There are two viewpoints to explain this event; nutritional deficiencies and releasing of inflammatory cytokines after severe WL.^2^^-^^4^

In this retrospective study, we reported four fascinating cases with definite diagnosis of MS presented after intended and intensive WL. These patients had been referred to “Nutrition Clinic” for consultation about WL. Here, we describe the past medical history of their WL diets and the short time intervals between WL and first presentation of MS. 

Case one was a 27-year-old woman with MS for over 7 years. Body mass index (BMI) was 29 at the time of going to clinic. She had a past history of obesity and usage of WL diets several times and recurrent weight gains from adolescence. The first presentation of MS was blurred vision due to optic neuritis. 2 months before presentation of this sign; she had lost 20 kg of her weight with an inappropriate WL diet. Owing to severe reduction in the amount of calorie consumed and the elimination of carbohydrate resources arbitrarily, she experienced 20 kg losing weight within 2 months (16 kg within the 1^st^ month and 4 kg within the 2^nd^ month). The mean of WL per month was 10 kg. She reported that she did not take any supplements and medications and had no exercise program.

Case two was a 32-year-old man with MS for over 6 years. BMI was 35 at the time of going to clinic. He had a past history of obesity since early adolescence and tried several unsuccessful WL diets. He had gone on a 6 months WL diet up to 1 month before the first presentation of MS. The first presentation of MS was blurred vision due to optic neuritis. He had lost 30 kg within the first 3 months. During the 3 months of continuing with the same WL diet, he had lost another 10 kg. The mean of WL per month was 6.7 kg.

Case three was a 30-year-old woman with MS for over 4 years. BMI was 33 at the time of going to clinic. The first presentation of MS was blurred vision due to optic neuritis. She had a past history of inappropriate WL diet just before the first presentation of MS without consuming any supplements or having any scheduled exercise programs. She had lost 13 kg within 45 days. The mean of WL per month was 8.6 kg. 

Case four was a 26-year-old woman diagnosed with MS for over 2 years. BMI was 28 at the time of going to clinic. The first presentation of MS was paresthesia of the lower limbs. She had a past history of several WL diets from the onset of adolescence. She had a hard dietary regimen without consuming any supplements or having any exercise programs just before the first presentation of MS. She lost 40 kg within 4 months. The mean of WL per month was 10 kg.

## Conclusion

Presented cases had experienced intended and intensive WL (mean of WL was 8.8 kg/month overall) in the closest time to expression of MS symptoms. They all had a history of obesity and made concerted efforts to reduce their weight with disordered and abnormal WL diets. We considered that severe or rapid WL with intensive food intake restriction may be etiologic or accelerating factor for MS due to nutritional deficiencies. In addition, inflammatory cytokines such as interferon-γ (IFN-γ), tumor necrosis factor-α, and interleukin-1 release due to intensive WL and massive lipolysis probably are involved in the incidence of MS.^3^^,^^4^ Our observations buttressed earlier studies suggesting that neurological adverse effects have been observed after severe WL in obese people.^4^

The bariatric surgery is one of the common ways of severe WL in which some nutritional deficiencies such as vitamin A, B, D, and E have been identified.^5^^,^^6^

Neurological complications affected both central and peripheral nervous system due to axonal loss and demyelination that observed in MS can occur after bariatric surgery through nutritional and inflammatory mechanisms.^4^^,^^6^

We express a new hypothesis in etiology of MS related to nutritional deficiencies and inflammatory processes accompanied severe WL. Further studies are recommended to investigate the accuracy of this hypothesis.

## Notes:

**How to cite this article:** Bitarafan S, Amani K, Sahraian MA, Sarraf P, Soltani D, Naser Moghadasi A, et al. The first attack of multiple sclerosis presented immediately after voluntary and intensive weight loss: A case series. Iran J Neurol 2017; 16(1): 41-2.
